# Subarachnoid extension and unfavorable outcomes in patients with supratentorial intracerebral hemorrhage

**DOI:** 10.1186/s12883-023-03087-9

**Published:** 2023-01-28

**Authors:** Jinjin Wang, Dandan Wang, Liheng Bian, Anxin Wang, Xiaoli Zhang, Ruixuan Jiang, Wenjuan Wang, Yi Ju, Jingjing Lu, Xingquan Zhao

**Affiliations:** 1 Department of Neurology, Beijing Tiantan Hospital, Capital Medical University, No.119 South 4th Ring West Road, Fengtai District Beijing, 100070 China; 2 China National Clinical Research Center for Neurological Diseases, Beijing, China; 3 Research Unit of Artificial Intelligence in Cerebrovascular Disease, Chinese Academy of Medical Sciences, Beijing, China; 4 Beijing Institute of Brain Disorders, Collaborative Innovation Center for Brain Disorders, Capital Medical University, Beijing, China

**Keywords:** Subarachnoid extension, Supratentorial intracerebral hemorrhage, Clinical outcomes, Survival incidence

## Abstract

**Objective:**

Our study aimed to investigate the association between the subarachnoid extension of intracranial hemorrhage (SAHE) and clinical outcomes in patients with supratentorial intracerebral hemorrhage (ICH).

**Methods:**

We analyzed the data from a prospective, multi-center, and registry-based database. Two experienced investigators independently assessed ICH imaging data. We compared baseline characteristics and follow-up outcomes. Multivariable logistic regression analysis was used to evaluate the association between SAHE and poor clinical outcomes. We also performed Kaplan–Meier curves and Cox proportional hazards regression analyses to analyze whether SAHE was relevant to a higher mortality rate.

**Results:**

A total of 931 patients were included in this study (SAHE vs. no SAHE, 121 [13.0%] vs. 810 [87.0%]). Patients with SAHE had more severe neurological deficits, higher scores of the mRS, and more remarkable mortality rates at follow-up (all *p* values < 0.05). In multivariable-adjusted models, SAHE was independently associated with a higher risk of poor outcomes (adjusted OR [95%CI]: 2.030 [1.142–3.608] at 3 months; 2.348 [1.337–4.123] at 1 year). In addition, SAHE remained an independent association with an increased death rate at 1 year (adjusted HR [95%CI], 1.314[1.057–1.635]). In the subgroup analysis, the correlation between SAHE and prognosis exists in patients with lobar or deep ICH.

**Conclusions:**

SAHE is independently associated with poor outcomes in patients with supratentorial ICH. It may provide a promising target for developing new predictive tools targeting ICH.

**Supplementary Information:**

The online version contains supplementary material available at 10.1186/s12883-023-03087-9.

## Introduction

Intracerebral hemorrhage (ICH) accounts for approximately 15% of stroke incidence but disproportionately 50% of stroke-related mortality worldwide [1–3]. About 2.89 million individuals die from hemorrhagic stroke each year [3–5]. Compared to ischemic stroke, there have not yet been proven effective therapies in definitely managing ICH, which causes substantial economic costs for acute treatment and post-stroke care [6, 7]. Thus, it is vital to understand relevant injury mechanisms to develop more accurate prognostic tools, which would contribute to major breakthroughs in therapeutic interventions.

Many markers based on computerized tomography (CT) are increasingly used for predicting hematoma enlargement (HE), ICH prognosis, and perihematomal edema (PHE), such as black hole signs, blend signs, and island signs [8, 9]. Several studies recently suggested that ICH patients with subarachnoid extension of hemorrhage (SAHE) seemed to have unfavorable outcomes [10, 11]. SAHE is defined as any extension of intracranial hematoma into the subarachnoid space, frequently observed following ICH [11]. Previous research proposed that blood component deposited in subarachnoid space also contributed to secondary injury after ICH, akin to aneurysmal subarachnoid hemorrhage (aSAH). However, only a tiny quantity of blood in ICH extends into subarachnoid space, compared to aSAH. Thus, it needs to be clarified how SAHE affects brain damage secondary to ICH.

Limited research focuses on the associations between SAHE and ICH prognosis, and related results remain controversial. Subgroup analysis from the INTERACT2 study found this association depended on a larger volume of ICH hematoma [10]. Recent results from the TICH-2 study demonstrated a predictive value of SAHE for early neurological deterioration in ICH patients [12]. Other studies may limit to a small sample and short-term follow-up. Thus, the underlying mechanisms of SAHE in ICH need to be further studied to illustrate the specific correlations and develop the aid decision-making tools. Since SAHE is more likely to occur in supratentorial ICH that accounts for a large amount of spontaneous ICH [11], patients with supratentorial ICH may be the key populations benefiting from targeting SAHE.

Therefore, our study aimed to primarily investigate whether SAHE was associated with short-term and long-term outcomes in patients with supratentorial ICH. Secondarily, we performed a subgroup analysis to determine the association between SAHE and prognosis in patients with lobar or deep ICH.

## Methods

### Study design and population

Our present study retrospectively analyzed the data from a prospective, multi-center, and registry-based database. Participants were consecutively recruited from 13 hospitals in Beijing from January 2014 to September 2016. The study was approved by the Institutional Review Board (IRB) of Beijing Tiantan Hospital (IRB No. KY2014-023–02). Informed consent was obtained from patients or their legal relatives before participating in this study. Researchers of the coordinating center finally summarized and analyzed all data.

The inclusion criteria of this study included: (1) supratentorial ICH diagnosed by the WHO standard and confirmed by non-contrast computed tomography (NCCT) images; (2) with age ≥ 18 years old; (3) arriving at emergency within 72 h after symptoms onset; (4) written consent was obtained. The exclusion criteria are as follows: (1) known major comorbidities or late-stage diseases, such as liver failure (Child–Pugh score C), end-stage kidney disease (estimated glomerular filtration rate [eGFR] < 15 ml/min per 1.73 m^2^, heart failure with reduced left ventricular ejection fraction [≤ 40%], and malignant tumor with a life expectancy of < 3 months) [13]; (2) ICH with secondary etiology, such as arteriovenous malformation, trauma, aneurysm, and coagulation disorders; (3) missing hematoma volumes or extreme values; (4) missing SAHE data or follow-up outcomes.

### Assessment of epidemiological data, medical history and other baseline data

All participants or their relatives performed a standardized questionnaire to obtain age, sex, medical history, drug uses, personal history, and other baseline data. Current smoking was defined as if a patient smoked more than one cigarette per day for at least one year, while current drinking was defined as if a patient had drunk more than ≥ 80 g of liquor daily for at least one year.

The Glasgow Coma Scale (GCS) and National Institute of Health Stroke Scale (NIHSS) scores were used to assess the baseline conscious status and neurological deficits. Admission blood pressure and other vital signs were also recorded. Blood samples were taken from the median cubital vein, and various blood indices were measured in the qualified clinical laboratories. We also noted if a patient underwent brain surgery during the hospital, including decompressive craniectomy, craniotomy evacuation of hematoma, hematoma aspiration, and lateral ventriculopuncture drainage.

### Assessment of imaging data and ICH data

**Fig. 1:**
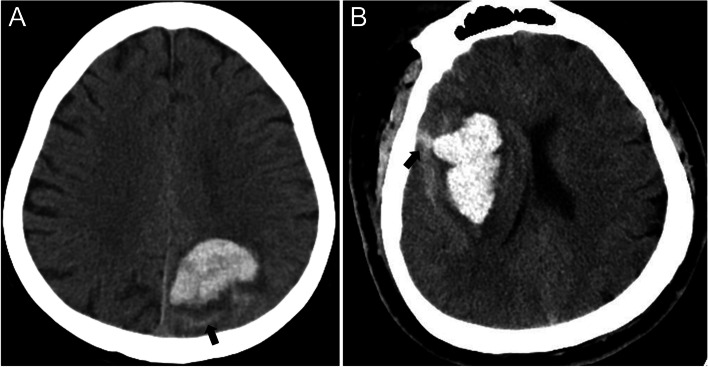
Intracerebral hemorrhage (ICH) with subarachnoid extension on baseline computed tomography. **A** Lobar ICH with subarachnoid extension (arrow). **B** Deep ICH with subarachnoid extension (arrow)

All patients underwent a baseline NCCT imaging scan according to standardized parameters. The coordinating center centrally gathered digital imaging in uncompressed DICOM formats. Two experienced investigators analyzed these imagings separately, who were blinded to other data. SAHE was defined as the presence of any blood in the subarachnoid space (Fig. 1). Furthermore, hematoma volume was calculated by the ABC/2 method. Once there was some uncertainty about the judgment of imaging data, other different experts would further determine the result.

### Follow-up and outcomes

The modified Rankin Scale (mRS) score was used to assess the functional status. The primary outcome was defined as an mRS score of 4–6, which usually represented a severe disability or death in ICH patients. A face-to-face interview was conducted at discharge to evaluate the NIHSS, GCS, and mRS scores. Telephone interviews were separately carried out at 1 month, 3 months, and 1 year after ICH symptoms onset. By telephone, patients or their relatives answered a structural questionnaire to determine their functional states. The death would be further recorded in detail. All investigators were well-trained and blinded to the patient’s baseline characteristics.

### Statistical analysis

The SAS software (version 9.4; SAS Institute, Cary, NC, USA) was used for statistical analysis. Continuous variables were shown as means ± standard deviation (SD) or medians (interquartile range, IQR). Student’s t-test or Kruskal–Wallis test compared them. Categorical variables were listed as counts (percentages, [%]), and we performed a Chi-square test or Fisher exact test to compare the baseline characteristics of the two groups. Next, we conducted univariable and multivariable logistic regression analyses to determine the association between SAHE and poor clinical outcomes. According to current literature, multivariable models adjusted the main baseline variables associated with SAHE and other variables related to ICH prognosis. Age and sex were adjusted in model 1. Comorbidities and ICH data were added in model 2. In model 3, baseline hematoma volume was adjusted based on model 2, which was considered independently related to the presence of SAHE [10]. Kaplan Meier (K-M) curves were used to estimate the overall survival probability within 1 month, 3 months, and 1 year of ICH onset, respectively. Cox proportional hazards regression analysis was used to describe and evaluate the correlation between SAHE and follow-up mortality rate. Finally, we performed a subgroup analysis stratified by age, gender, and hematoma locations. All reported p-values are two-sided, and the *p*-value < 0.05 was considered the threshold of statistical significance.

## Results

**Fig. 2:**
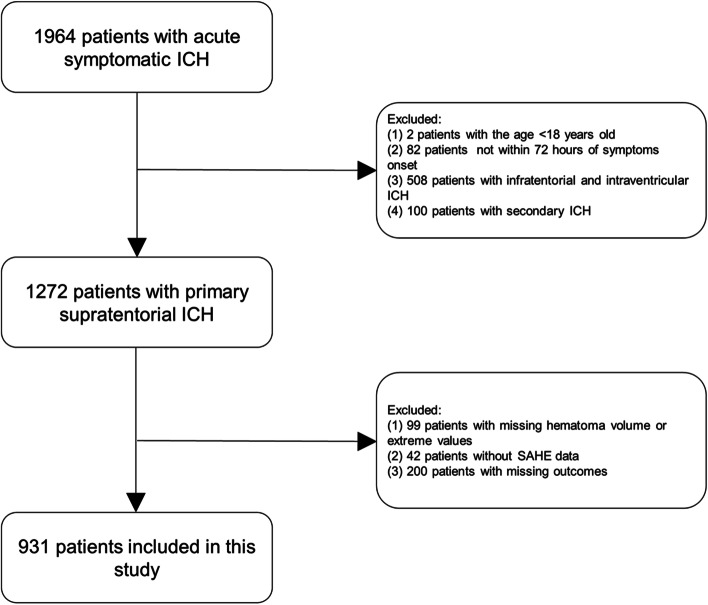
The flowchart of our present study

A total of 931 patients were included in our study after excluding 1033 patients (Fig. 2). There were 629 males and 302 females, with an average of 58.9 ± 12.6 years old. According to the existence of SAHE or not, all patients were divided into two groups (121 [13.0%] vs. 810 [87.0%]). SAHE was identified with good interrater reliability (kappa = 0.80). Baseline characteristics are presented in Table 1. Compared to those without SAHE, patients with SAHE tended to have a much more severe neurological deficit (17 [9-27] vs. 10 [4-16], *p* < 0.0001) and a significantly larger baseline hematoma volume (46.3 [21.8–61.5] vs. 13.9 [6.0–29.3], *p* < 0.0001). There was a much higher proportion of lobar hemorrhage and intraventricular extension in the patients with SAHE (*p* values < 0.05). We also found much higher levels of C-reactive protein (CRP) and significantly higher white blood cell (WBC) counts in patients with SAHE (*p* values < 0.05). In addition, a higher percentage of patients with SAHE underwent related brain surgery (*p* < 0.05). There was no significant difference in age, gender, ethnic distribution, and the proportions of smokers and drinkers between the two groups. Besides, no difference was noticed in the percentage of comorbidities, anticoagulants, and antiplatelet drug uses.

**Table 1:** Baseline characteristics of study participants with SAHE and without SAHE

Characteristics	Study sample (*N* = 931)	With SAHE (*N* = 121)	Without SAHE (*N* = 810)	*P* Value
Age (years)^a^	58.9 ± 12.6	60.7 ± 13.7	58.7 ± 12.5	0.0505
Male^c^	629 (67.6%)	76 (62.8%)	553 (68.3%)	0.2313
Ethnic Han^c^	882 (94.7%)	111 (92.7%)	771 (95.2%)	0.2801
Current smoking^c^	308 (33.1%)	38 (31.4%)	270 (33.3%)	0.0939
Current drinking^c^	342 (36.7%)	44 (36.4%)	298 (36.8%)	0.5926
Hypertension^c^	672 (72.3%)	80 (66.7%)	592 (73.1%)	0.1427
Diabetes mellitus^c^	137 (14.7%)	18 (14.9%)	119 (14.7%)	0.9573
Dyslipidemia^c^	91 (9.8%)	12 (9.9%)	79 (9.8%)	0.9547
Prior cerebral infarction^c^	145 (7.4%)	19 (15.7%)	126 (15.6%)	0.9668
Prior antiplatelet use^c^	157 (16.9%)	20 (16.5%)	137 (16.9%)	0.7229
Prior anticoagulant use^c^	11 (1.2%)	2 (1.7%)	9 (1.1%)	0.8744
Admission SBP (mmHg)^a^	168 ± 27	171 ± 29	167 ± 27	0.2417
Admission DBP (mmHg)^a^	97 ± 18	96 ± 19	97 ± 18	0.4229
Admission GCS^b^	14 (10–15)	10 (6–14)	14 (11–15)	< 0.0001
Admission NIHSS^b^	10 (5–18)	17 (9–27)	10 (4–16)	< 0.0001
Admission CRP (mg/L)^b^	5.6 (1.6–21.4)	15.1 (3.7–41.4)	5.0 (1.4–16.1)	0.0492
Admission WBC (10^9^/L)^b^	8.8 (6.8–11.7)	11.3 (8.4–15.2)	8.5 (6.7–11.3)	< 0.0001
Location of hematoma^c^				< 0.0001
Lobar	252 (27.1%)	65 (53.7%)	187 (23.1%)	
Deep	679 (72.9%)	56 (46.3%)	623 (76.9%)	
Intraventricular extension^c^	325 (34.9%)	72 (59.5%)	253 (31.2%)	< 0.0001
Hematoma volume (ml)^b^	15.8 (7.3–37.0)	46.3 (21.8–61.5)	13.9 (6.0–29.3)	< 0.0001
Surgical treatment^c^	188 (20.2%)	39 (32.2%)	149 (18.4%)	0.0004

*SAHE* extension of hemorrhage into the subarachnoid space, *SBP* systolic blood pressure, *DBP* diastolic blood pressure, *GCS* Glasgow Coma Scale, *NIHSS* National Institutes of Health Stroke Scale, *CRP* C-reactive protein*, WBC* white blood cell

^a^Continuous variables are expressed as means ± (SD) or ^b^medians (IQR) according to normal distribution

^c^Category variables are expressed as number (%)

**Fig. 3:**
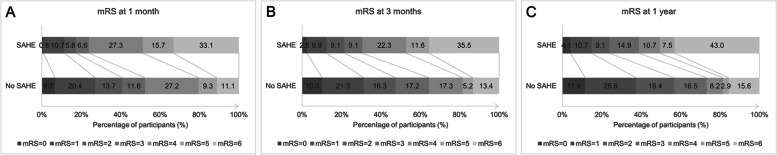
The distribution of modified Rankin Scale (mRS) scores between two groups with SAHE and without SAHE. **A** at 1 month; **B** at 3 months; **C** at 1 year

We also compared the clinical outcomes of the two groups. Patients with SAHE had a higher score of the mRS at each time-point (all *p* values < 0.05, Table 2). The distribution of mRS score is shown in Fig. 3. In patients with SAHE, there was a higher death rate at follow-up (all *p* values < 0.05). In addition, the proportion of severe disability was significantly higher in the SAHE group (all *p* values < 0.05).

**Table 2:** Associations between ICH prognosis and different groups with SAHE and without SAHE

Outcomes	Study sample	With SAHE	Without SAHE	*P* Value
	**(** ***N*** ** = 931)**	**(** ***N*** ** = 121)**	**(** ***N*** ** = 810)**	
**At 1 month**				
mRS score^a^	4 (1–4)	4 (4–6)	3(1–4)	< 0.0001
Death^b^	129 (13.9%)	40 (33.1%)	89 (11.0%)	< 0.0001
Severe disability^b^	348 (43.4%)	52 (64.2%)	296 (41.1%)	< 0.0001
Death or severe disability^b^	477 (51.2%)	92 (76.0%)	385 (47.5%)	< 0.0001
**At 3 months**				
mRS score^a^	3 (1–4)	4 (3–6)	3(1–4)	< 0.0001
Death ^b^	143 (15.4%)	43 (35.5%)	100 (12.4%)	< 0.0001
Severe disability^b^	223 (28.7%)	41 (52.6%)	182 (25.6%)	< 0.0001
Death or severe disability^b^	366 (39.4%)	84 (69.4%)	282 (34.9%)	< 0.0001
**At 1 year**				
mRS score^a^	3 (1–4)	5 (3–6)	2(1–4)	< 0.0001
Death^b^	178 (19.1%)	52 (43.0%)	126 (15.6%)	< 0.0001
Severe disability^b^	112 (14.9%)	22 (31.9%)	90 (13.2%)	< 0.0001
Death or severe disability^b^	290 (31.2%)	74 (61.2%)	216 (26.7%)	< 0.0001

*ICH* intracerebral hemorrhage, *SAHE* extension of hemorrhage into the subarachnoid space, *mRS* the modified Rankin Scale

^a^Continuous variables are expressed as medians (IQR) according to normal distribution

^b^Category variables are expressed as number (%)

Then, we found that SAHE was associated with an increased risk of poor functional outcomes (all *p* values < 0.05). After adjusting age and sex, a similar relevance was observed between SAHE and severe disability or death at follow-up (all *p* values < 0.05). This independent association also remained in model 2, added by other factors. However, after further adjusting hematoma volume in model 3, this relevance between SAHE and poor prognosis just existed at 3 months and 1 year (adjusted OR [95%CI]: 2.030 [1.142–3.608] at 3 months, 2.348 [1.337–4.123] at 1 year). The details are listed in Table 3.

**Table 3:** Univariate and multivariate-adjusted OR and 95% CI for severe disability or death (mRS score = 4–6) according to the presence of SAHE

Outcomes	Events	Crude		Model 1		Model 2		Model 3	
	**N (%)**	**OR (95%CI)**	***P*** ** Value**	**OR (95%CI)**	***P*** ** Value**	**OR (95%CI)**	***P*** ** Value**	**OR (95%CI)**	***P*** ** Value**
At 1 month	92 (76.0%)	3.502 (2.256–5.435)	< 0.0001	3.433 (2.198–5.360)	< 0.0001	2.011 (1.098–3.682)	0.0236	1.501 (0.810–2.782)	0.1968
At 3 months	84 (69.4%)	4.243 (2.808–6.411)	< 0.0001	4.244 (2.776–6.487)	< 0.0001	2.537 (1.445–4.454)	0.0012	2.030 (1.142–3.608)	0.0158
At 1 year	74 (61.2%)	4.330 (2.910–6.442)	< 0.0001	4.568 (2.984–6.992)	< 0.0001	2.768 (1.593–4.810)	0.0003	2.348 (1.337–4.123)	0.0030

Model 1: adjusted for age and gender

Model 2: adjusted for age, gender, hypertension, diabetes mellitus, prior ischemic stroke, dyslipidemia, current drinking, current smoking, admission GCS, admission NIHSS, admission hematoma location, and intraventricular extension

Model 3: adjusted for age, gender, hypertension, diabetes mellitus, prior ischemic stroke, dyslipidemia, current drinking, current smoking, admission GCS, admission NIHSS, admission hematoma location, intraventricular extension, and admission hematoma volume

*SAHE* extension of hemorrhage into the subarachnoid space, *OR* odds ratio, *CI* confidence interval

**Fig. 4:**
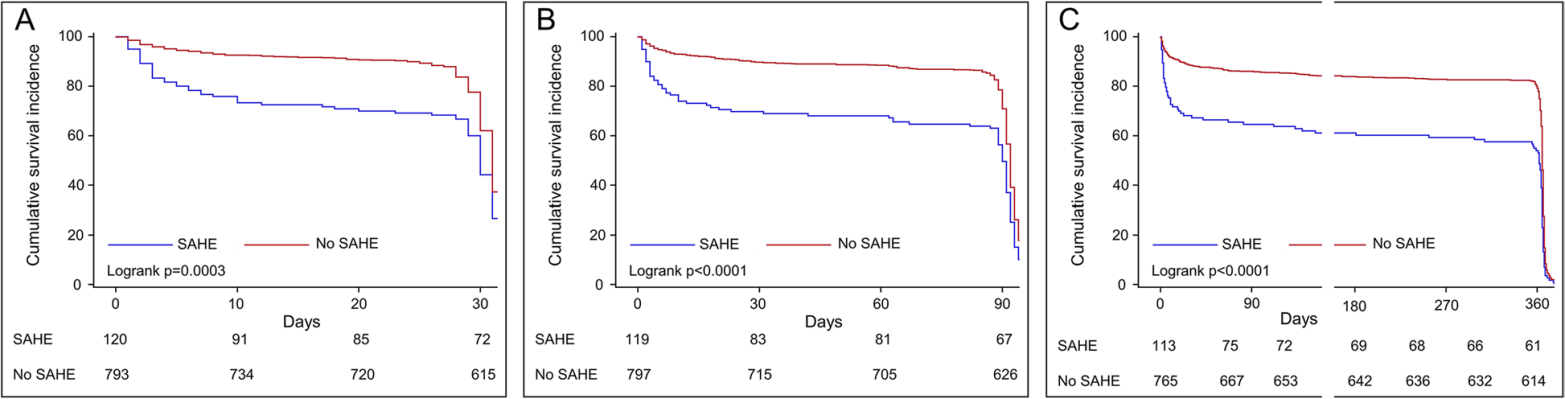
The cumulative survival rate of all patients between two groups with SAHE and without SAHE. **A** at 1 month; **B** at 3 months; **C** at 1 year

A Kaplan–Meier curve of all participants was also made, with a total survival probability as cumulative incidence. The patients with SAHE had significantly lower survival rates at follow-up (all *p* values < 0.05, Fig. 4). After adjusting relevant hazard risks in model 1 and model 2, the association still existed between SAHE and an increased mortality rate (all *p* values < 0.05). In model 3, SAHE was independently correlated to an elevated mortality rate at 1 year (adjusted HR[95%CI], 1.314[1.057–1.635]). Nevertheless, we did not oberserve the correlation at 1 month and 3 months (Table 4).

**Table 4:** Univariate and multivariate-adjusted HR and 95% CI for death according to the presence of SAHE

Outcomes	Events	Crude		Model 1		Model 2		Model 3	
	**N (%)**	**HR (95%CI)**	***P*** ** Value**	**HR (95%CI)**	**P Value**	**HR (95%CI)**	***P*** ** Value**	**HR (95%CI)**	***P*** ** Value**
At 1 month	40 (33.1%)	1.369 (1.129–1.659)	0.0014	1.370 (1.129–1.662)	0.0014	1.096 (0.889–1.353)	0.3907	1.074 (0.866–1.332)	0.5166
At 3 months	43 (35.5%)	1.427 (1.177–1.731)	0.0003	1.428 (1.176–1.734)	0.0003	1.116 (0.903–1.378)	0.3093	1.074 (0.867–1.331)	0.5153
At 1 year	52 (43.0%)	1.651(1.354–2.014)	< 0.0001	1.635 (1.340–1.994)	< 0.0001	1.343 (1.084–1.664)	0.0070	1.314 (1.057–1.635)	0.0140

Model 1: adjusted for age and gender

Model 2: adjusted for age, gender, hypertension, diabetes mellitus, prior ischemic stroke, dyslipidemia, current drinking, current smoking, admission GCS, admission NIHSS, admission hematoma location, and intraventricular extension

Model 3: adjusted for age, gender, hypertension, diabetes mellitus, prior ischemic stroke, dyslipidemia, current drinking, current smoking, admission GCS, admission NIHSS, admission hematoma location, intraventricular extension, and admission hematoma volume

*SAHE* extension of hemorrhage into the subarachnoid space, *HR* hazard ratio, *CI* confidence interval

**Fig. 5:**
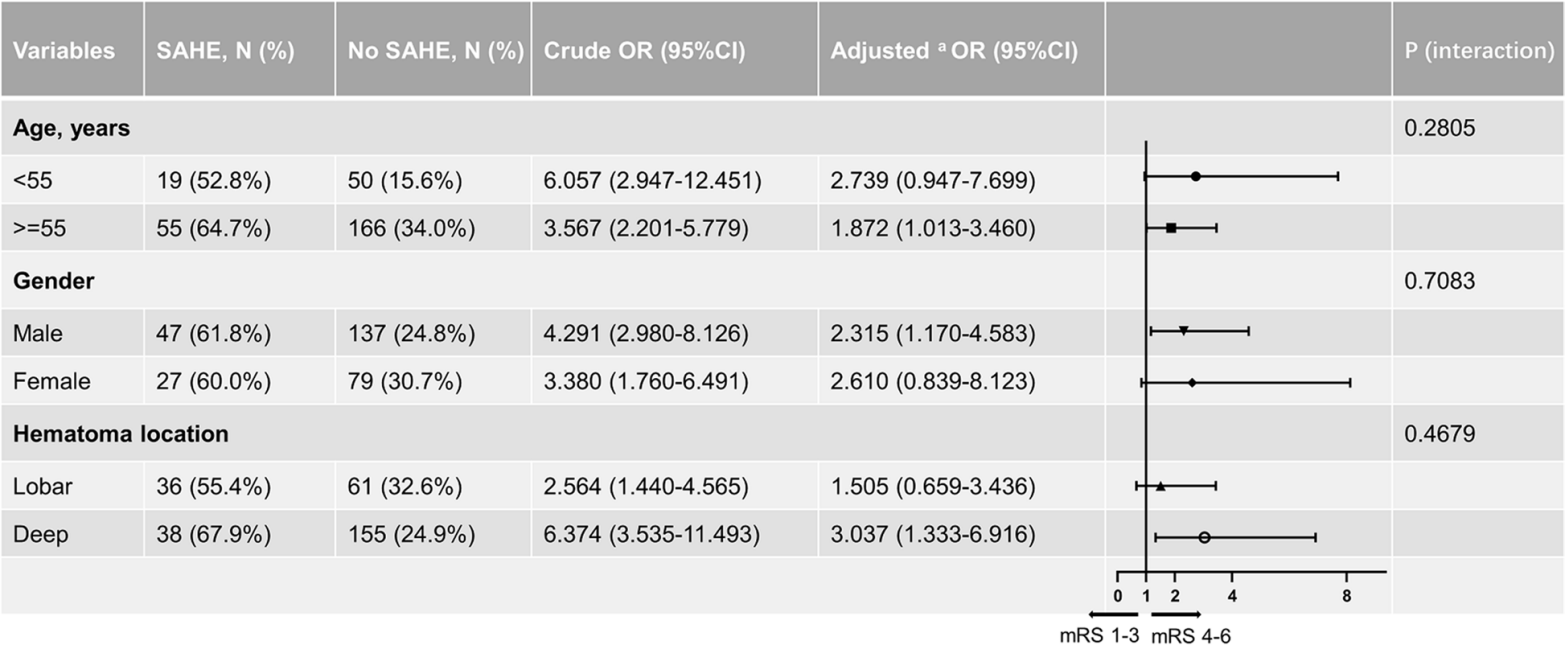
Subgroup analysis about the association between SAHE and 1-year poor outcomes according to age, gender, and hematoma locations. ^a^Adjusted model was adjusted for age, sex, hypertension, diabetes mellitus, prior ischemic stroke, dyslipidemia, current drinking, current smoking, admission GCS, admission NIHSS, admission hematoma location, intraventricular extension, and admission hematoma volume. *SAHE*, extension of hemorrhage into the subarachnoid space; *OR*, odds ratio; CI, confidence interval

Further subgroup analysis was plotted to examine the efffects of SAHE according to age, sex, and the locations of admission hematoma. The results showed no significant interaction between the SAHE and age, gender, or ICH location group, respectively (Fig. 5).

## Discussion

In patients with primary supratentorial ICH, this study suggests that: (1) SAHE is associated with more severe baseline neurological deficits and poorer prognosis at follow-up; (2) SAHE is independently correlated to an increased risk of unfavorable outcomes at 3 months and 1 year; (3) there is an independent association between SAHE and a higher risk of death at 1 year; (4) no significant interaction was found between SAHE and age, gender, or hematoma locations, respectively. The results indicate that SAHE may serve as a predictive marker, especially in patients with mild to moderate ICH.

Our main finding is the association between SAHE and clinical outcomes in patients with supratentorial ICH, including short-term and long-term prognosis. In previous studies, patients with SAHE account for 7.0%-39.7% of all included participants due to the difference in antithrombotic drugs, hematoma locations, and baseline hematoma volumes [10, 11], whereas the percentage of SAHE was 13.0% in our study. Unlike Matthew’s results [11], our study had more young patients, fewer taking anti-platelet or anti-coagulant drugs, and fewer with lobar ICH. They have found that SAHE was associated with a higher risk of 14-day death and a higher mRS score at 28 days, even after adjusting the ICH score. Our findings are partly in line with it. We also found the relevance between SAHE and higher scores of mRS and an increased risk of mortality at 1 month. However, our results did not suggest this association between SAHE and acute prognosis after adjusting the admission hematoma volume. The result is consistent with one subgroup analysis from the INTERACT2 study, which showed that this association of SAHE depended on larger hematoma volumes [10]. Based on these studies, we further discussed the relationship between SAHE and long-term prognosis in patients with supratentorial ICH. Our results showed that SAHE was independently associated with composite severe disability or death at 3 months and 1 year. Moreover, our analyses indicated that patients with SAHE had a higher risk of 1-year death. All these results suggest an correlation between ICH prognosis and SAHE on admission.

Although it is still unclear for the association between SAHE and ICH prognosis, several mechanisms might help explain it. Firstly, SAHE usually occurs along with a much larger hematoma, as shown in previous studies and our results [10, 11]. It is widely recognized that baseline hematoma volume is an independent factor for hematoma enlargement and poor prognosis in patients with ICH [14, 15], especially in the acute stage. On the one hand, a larger hematoma volume would cause the compression of surrounding brain tissue and aggravate neurological deficits. On the other hand, a larger hematoma volume would contribute to more severe secondary injury triggered by a cascade of events such as inflammation [16]. Recently, a study reported that the effects of ICH volume decreased over time that our study also proved [15]. In the early stage of ICH, SAHE was associated with poor outcomes and the relationship did not exist after adjusting the hematoma volume. Nevertheless, the dependence weakened at 3 months and 1 year, even after adjusting ICH severity measures and ICH volume. Secondly, there is some evidence that SAHE would evolve into cortical superficial siderosis (cSS) [17], relevant to increased risks of lobar ICH and recurrent ICH [18, 19]. Tsai recently reported that the cSS was an independent predictor for vascular events even in mixed ICH, including cerebral amyloid angiopathy (CAA) and deep hypertensive ICH. Pathologically, SAHE might be an indicator of vulnerable vessels and thus presents a status of cerebral small vessel disease underlying the acute ICH [20–22]. Thirdly, SAHE may contribute to cerebral vasospasm and ensuing delayed cerebral ischemia (DCI), and further decelerating the recovery post-ICH. Platz observed that DCI were more likely to be suffered in patients with aneurysmal subarachnoid hemorrhage (SAH) and an additional lobar hematoma [23]. However, related research is limited; and thus, this aspect warrants further confirmation in spontaneous ICH. In addition to mentioned causes above, the relationship between SAHE and complications presumably provoked injury and worsen outcomes after primary ICH,, such as fever and seizures [24, 25]. Additionally, the association between SAHE and severer inflammatory response may be involved in the injury process after ICH, which is also noticed in our results [26].

Recent research reported a prognostic value of SAHE for hematoma enlargement in lobar ICH rather than deep ICH [27]. In this present study, the subgroup analysis has indicated no discrepancy in the distribution of hematoma locations. Since SAHE occurs much more frequently in lobar ICH rather than deep ICH (25.8% vs. 8.2% in our study), the results may also provide more about deep ICH, such as severer conditions and higher hypertensive small vessel disease burdens [28–30].

There are several strengths in our methods and results. The main strengths include a prospective and multi-center design, a large sample, and a continuous follow-up until 1 year after ICH. More importantly, our study may have some implications for prognosis prediction and clinical management. In this study, SAHE is defined by CT imaging and may provide an easily obtained, low-cost, and reliable imaging marker for developing prognostic tools of ICH. Patients with SAHE may have an increased risk of poor functional outcomes. Thus, it is more applicable to monitoring conditions in the emergency room, especially in some hospitals with limited resource availability. Moreover, these patients need to be paid more attention to prevent deterioration and manage risk factors, such as blood pressure and infections. Besides, the distinction of locations of ICH is one of our novelties, which may help identify more accurate markers for predicting ICH outcomes. As more researchers focus on SAHE, further research is warranted to investigate whether the combination of SAHE and other biomarkers can improve the prognostic accuracy of current prediction models.

However, there are a few limitations that we have to admit in our study. Firstly, this study included ICH patients with a median hematoma volume of 15.8 (7.3–37.0) ml; thus, our results are mainly applied to patients with mild to moderate ICH. Secondly, we calculated the hematoma volume by the traditional method of ABC/2 rather than using a more accurate method by the semi-automatic software. Third, the number of patients with SAHE is relatively small that may lead to the possible overfitting of regression models, so we used multiple models to avoid it as much as possible. In addition, there are no magnetic resonance imaging (MRI) data to discuss related secondary infarcts caused by post-ICH vasospasm, which may contribute to clarifying the inner mechanisms. Finally, SAHE was not further classified into convexity SAH or other types of SAH because recent studies have reported the prognostic utility of convexity SAH in patients with CAA [31]. Future studies will discuss the association between different types of SAHE and ICH prognosis in various etiologies, combining NCCT and further MRI data.

## Conclusions

In conclusion, our present study suggests that SAHE is independently associated with poor outcomes in patients with supratentorial ICH. It should be noted that the association still exists in lobar or deep ICH subgroup. This study provides evidence for the prognostic value of SAHE in patients with ICH. More studies must be conducted to clarify the mechanisms and guide effective treatment decisions.

## Supplementary Information

**Additional file 1: Supplementary table 1.** Univariate and multivariate-adjusted OR and 95% CI for severe disability or death (mRS score = 4-6) according to the presence of SAHE. **Supplementary table 2.** Univariate and multivariate-adjusted HR and 95% CI for follow-up death according to the presence of SAHE.

## Data Availability

The datasets used and/or analysed during the current study are available from the corresponding author on reasonable request.
