# Supplementary material for: Subarachnoid extension and unfavorable outcomes in patients with supratentorial intracerebral hemorrhage
Source: BMC Neurol. 2023 Jan 28;23:46. doi: 10.1186/s12883-023-03087-9 (PMC9883933; doi:10.1186/s12883-023-03087-9)
Supplement: Supplementary file 1 — Additional file 1: Supplementary table 1. Univariate and multivariate-adjusted OR and 95% CI for severe disability or death (mRS score = 4-6) according to the presence of SAHE. Supplementary table 2. Univariate and multivariate-adjusted HR and 95% CI for follow-up death according to the presence of SAHE. [file 12883_2023_3087_MOESM1_ESM.docx]

**Supplementary table 1.** Univariate and multivariate-adjusted OR and 95% CI for severe disability or death (mRS score = 4-6) according to the presence of SAHE.

| **Outcomes** | **Events**  **N (%)** | **Model 4** | |
| --- | --- | --- | --- |
|  |  | **OR (95%CI)** | **P Value** |
| At 1 month | 92 (76.0%) | 1.621 (0.851-3.089) | 0.1419 |
| At 3 months | 84 (69.4%) | 2.126 (1.166-3.879) | 0.0139 |
| At 1 year | 74 (61.2%) | 2.674 (1.487-4.809) | 0.0010 |

Model 4: adjusted for age, gender, hypertension, diabetes mellitus, prior ischemic stroke, dyslipidemia, current drinking, current smoking, admission GCS, admission NHISS, admission hematoma location, intraventricular extension, admission hematoma volume, and blood glucose.

*SAHE*, extension of hemorrhage into the subarachnoid space; *OR,* odds ratio; *CI*, confidence interval.

**Supplementary table 2.** Univariate and multivariate-adjusted HR and 95% CI for follow-up death according to the presence of SAHE.

| **Outcomes** | **Events** | **Model 4** | |
| --- | --- | --- | --- |
|  | **N (%)** | **HR (95%CI)** | **P Value** |
| At 1 month | 40 (33.1%) | 1.049 (0.841-1.310) | 0.6700 |
| At 3 months | 43 (35.5%) | 1.046 (0.836-1.308) | 0.6948 |
| At 1 year | 52 (43.0%) | 1.322 (1.056-1.654) | 0.0147 |

Model 4: adjusted for age, gender, hypertension, diabetes mellitus, prior ischemic stroke, dyslipidemia, current drinking, current smoking, admission GCS, admission NHISS, admission hematoma location, intraventricular extension, admission hematoma volume, and blood glucose.

*SAHE*, extension of hemorrhage into the subarachnoid space; *HR,* hazard ratio; *CI*, confidence interval.
